# Thermal Conductivity of Graphene-Polymer Composites: Mechanisms, Properties, and Applications

**DOI:** 10.3390/polym9090437

**Published:** 2017-09-15

**Authors:** An Li, Cong Zhang, Yang-Fei Zhang

**Affiliations:** Department of Materials Science and Engineering, College of Engineering, Peking University, Beijing 100871, China; lian1993@pku.edu.cn (A.L.); 1601214778@pku.edu.cn (C.Z.)

**Keywords:** graphene, polymer composites, thermal conductivity, mechanisms, properties, applications

## Abstract

With the integration and miniaturization of electronic devices, thermal management has become a crucial issue that strongly affects their performance, reliability, and lifetime. One of the current interests in polymer-based composites is thermal conductive composites that dissipate the thermal energy produced by electronic, optoelectronic, and photonic devices and systems. Ultrahigh thermal conductivity makes graphene the most promising filler for thermal conductive composites. This article reviews the mechanisms of thermal conduction, the recent advances, and the influencing factors on graphene-polymer composites (GPC). In the end, we also discuss the applications of GPC in thermal engineering. This article summarizes the research on graphene-polymer thermal conductive composites in recent years and provides guidance on the preparation of composites with high thermal conductivity.

## 1. Introduction

Thermal management has become a crucial issue in the modern electronics industry as electronic devices have become more integrated and miniaturized. The power required for some processor modules can reach 250 W in a high-performance computer, leading to heat loads as large as 1 kW in this system [1]. If the heat can-not be dissipated promptly, the lifetime and the efficiency of the system could be reduced, or even breakdown. In this situation, materials with high thermal conductivity are strongly needed to dissipate the heat and solve the problem [2].

Polymers have a lot of advantages, such as being lightweight, low cost, easy to process, and exhibiting good corrosion resistance. However, most polymers are heat insulators and have a thermal conductivity between 0.1 and 0.5 W m^−1^ K^−1^ [3], which is due to their amorphous state. There are three kinds of carriers in solids to transport energy: phonons, electrons, and photons [4]. Phonons are quantized modes of vibration in a rigid crystal lattice, which is the fundamental mechanism of heat conduction in most polymers. Polymers in amorphous state are usually considered to have lots of defects that contribute to numerous phonon scatting, leading to a low thermal conductivity [5].

In past years, a lot of works have studied thermal conductive polymer-based composites. Many different materials with high thermal conductivity have been used as fillers to improve the thermal conductivity of composites, such as boron nitride (BN) [6,7,8,9], carbon nanotubes (CNTs) [10,11,12,13,14], aluminum oxide [15,16,17], diamond [18,19,20,21], and graphene [22,23].

Graphene has attracted great attention because of its unique two dimensional (2D) structure and novel properties, such as the zero-gap band structure, high electron mobility, and high thermal conductivity [24]. Balandin and his co-workers reported a measurement of the thermal conductivity of suspended single-layer graphene around 5000 W m^−1^ K^−1^, which was one of the highest thermal conductivities of currently known materials [25]. Although there are lots of reviews on the thermal conductivity of polymer-based composites, system summaries on thermal conductive graphene-polymer composites are rare [2,3,4]. In this situation, it is necessary to review the advances in the thermal conductivity of graphene-polymer composites.

In this article, we review the advances in thermal conductivity of graphene-polymer composites in recent years. Special attention is given to the mechanism, the properties, and the influence factors of graphene-polymer composites. Additionally, we discuss the applications of thermal conductive graphene-polymer composites.

## 2. Thermal Conductive Mechanisms

### 2.1. Thermal Conductive Mechanisms in Graphene

**Figure 1:**
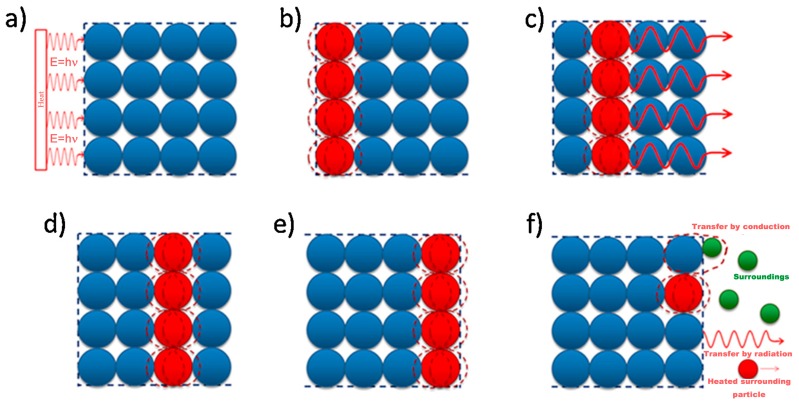
The schematic of thermal conductance in a crystalline material [29]. Copyright (2016), with permission from Elsevier.

In solid materials, heat is carried by phonons and electrons [26]. In metals, thermal conductivity is due to free carriers of electrons. Copper is a good thermal conductor with a thermal conductivity of 400 W m^−1^ K^−1^ at room temperature, and the attribution from phonons is limited to 1–2% of the total [27]. The thermal conductivity of graphene is attributed to phonons and electrons because of its metallic property [28]. However, the contribution of electrons to the thermal conductivity of graphene is relatively rare. In general, it is believed that the thermal conductivity of graphene is mainly accomplished by phonons [27]. Figure 1 is a schematic of heat conduction in crystalline materials, which can also be applied to graphene [29]. When one side of the crystal lattice makes contact with the heat source, heat conducts to the first layer atoms in the form of vibrations. Due to the dense packing of atoms in the lattice and the strong chemical bonds between them, the vibrations of the first layer atoms quickly spread to the neighboring atoms, and the neighboring atoms pass the vibrations to the other neighboring atoms, which results in rapid heat transfer in crystalline materials. In graphene, which has the ideal structure, all of the carbon atoms are fixed by a covalent bond to a layer. When some of the atoms in the graphene come into contact with the heat source and begin to vibrate, the vibrations will quickly pass to the surrounding atoms by the strong force of the covalent bond. In other words, the heat transfers from one position to another in graphene. In some studies, the researchers believe that the heat in graphene is transferred by the form of phonon waves, and some researchers have detected and proved this speculation [30,31,32]. In fact, most of the graphene used to manufacture the thermal conductive composites is multilayer graphene, such as graphene nanosheets and graphene nanoplatelets. When one of the layers in multilayer graphene begins to vibrate, due to the weak force of the van der Waals force between each layer, vibrations are difficult to pass on to the adjacent graphene layers. That is, heat is difficult to transfer through the interlayer of graphene. As a result, anisotropic heat conduction exists in the multilayer graphene. This phenomenon has been proved by many researches [25,33,34,35,36].

### 2.2. Thermal Conductive Mechanisms in Polymers

**Figure 2:**
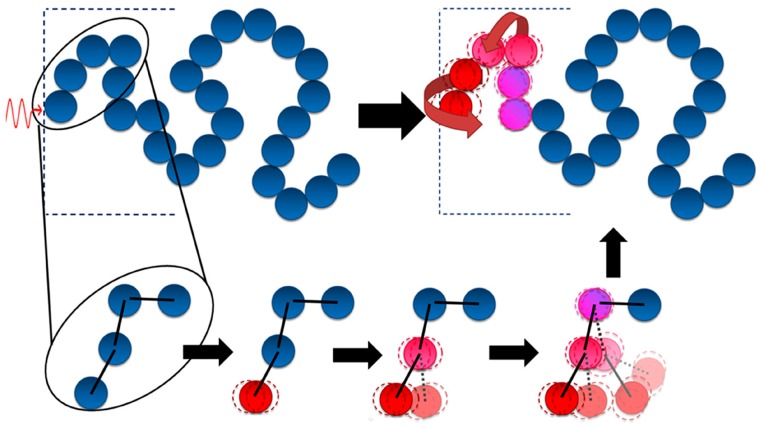
Schematic of thermal conductive mechanisms in polymer [29]. Copyright (2016), with permission from Elsevier.

Thermal conduction through a polymer is a complicated process, which is influenced by many parameters like crystallinity, temperature, orientation of the macromolecules, and so on [37,38,39]. Phonons are usually considered to be thermal carriers in polymers because there is a mere free electron [40]. Burger and her colleagues discussed the mechanism of heat transfer in an amorphous polymer and described it using a schematic diagram, which is presented in Figure 2 [29]. When the surface of the polymer makes contact with the heat source, heat transfers to the first atom of the molecular chain in the form of a vibration, then the nearest atom, and then the next. Heat will not propagate as a wave, like in graphene, but diffuse slower. Heat transfer in a molecular chain will also cause the disordered vibration and rotation of atoms, which significantly reduces the thermal conductivity of the polymer. A good conductor has a complete lattice structure, and atoms accumulate closely. When heat reaches the first atom, it will quickly transfer to the last one. However, heat transfer in a bad conductor causes the vibration and rotation of atoms, which will significantly reduce the thermal conductivity [29].

### 2.3. Thermal Conductive Mechanisms in Graphene-Polymer Composites

**Figure 3:**
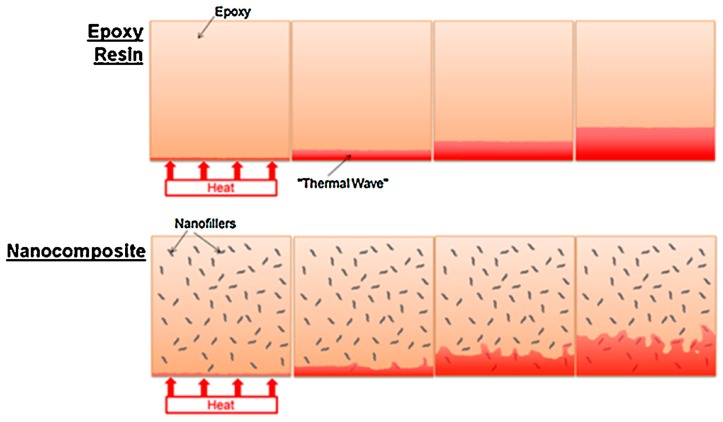
Thermal conductivity by graphene in a graphene-polymer composite [29]. Copyright (2016), with permission from Elsevier.

The thermal conductive mechanism of graphene in polymers is more complex. In general, graphene has a very high specific surface area. When being added in a polymer, large numbers of interfaces are produced [41]. These interfaces will lead to phonon scattering and introduce ultrahigh interfacial thermal resistance. Therefore, it is difficult for heat to transfer through the graphene-polymer interface [42]. There is much research discussing the thermal conductive mechanisms in the interface of graphene-polymer composites [2,3,4,29]. Since mismatches between graphene and the polymer exist, the interface will result in phonon scattering and hinder the heat transfer [43]. For example, supposing that during the same time of Δt, the heat transfers from one side of the graphene to the other. But in the polymer, the heat passes over a very short distance attributed to phonon scattering. When the loading of the filler is below the percolation threshold, the fillers cannot connect to each other to form a thermal conduction pathway. In this case, the interfacial thermal resistance of graphene and the polymer will be the main factor determining the thermal conductivity of the composite. Surface modification of the graphene has been proved to be an applicable method to enhance graphene-polymer interface interaction, and an efficient technique to decrease interfacial thermal resistance. In a composite, graphene acts as a highly thermal conductive channel, while the modified surface affords covalent and non-covalent bonding with the molecular chains of the polymer matrix, which will facilitate the phonon transfer from the graphene to the polymer and also promote the phonon transfer from the polymer to the graphene [44]. In many studies, researchers have considered that the molecular chains of polymer and the molecular chains on the surface of graphene can intertwine with each other and form an interlayer. This interlayer will decrease the interfacial phonon scattering and minimize the interface thermal resistance by intertwined molecular chains [45,46]. However, when the loading is above the percolation threshold, the heat in the composite mainly transfers through the thermal conduction pathway, due to the high thermal conductivity of graphene. Figure 3 shows the case of graphene acting as an efficient thermal conduction channel. In the course of time Δt, the heat could transfer over a longer distance in graphene than the polymer matrix. When composites make contact with the heat source, heat transfers though graphene very quickly, which will increase the thermal conductivity. Increasing the number of thermal pathways and reducing the thermal resistance between graphene and the graphene-polymer interface are recommended steps for preparing a composite with high thermal conductivity [3].

## 3. Recent Advances in Thermal Conductivity of Graphene-Polymer Composites

In recent years, more studies have been using graphene and its derived materials to prepare thermal conductive composite materials. The morphology of graphene in the polymer matrix significantly affects the thermal conductivity of the composites [2]. In this section, we review the advances in thermal conductivity of graphene-polymer composites. To review them more systematically, this section is divided into two parts, according to the different morphology of graphene in the polymer matrix. In the first part, the random dispersion of graphene in yjr polymer matrix is discussed. Random dispersion refers to the addition of graphene to the matrix, which is performed by a simple method, such as agitation, sonication, and blending, etc. Besides, there is no special method employed to control the orientation of graphene in matrix. In the second part, we discuss graphene with a specific orientation in the polymer matrix. This refers to the unusual structures of graphene in the polymer matrix, including the orientation, three-dimension structure (3D), and separate structure, etc. The term “graphene-related materials” is used to refer to the materials associated with graphene, which have different names in different literatures. These include graphene nanosheets, graphene nanoplatelets, graphene sheets, graphene flakes, graphene film, reduced graphene oxides, and graphene foam, etc.

### 3.1. Graphene with Random Orientation in the Polymer Matrix

**Figure 4:**
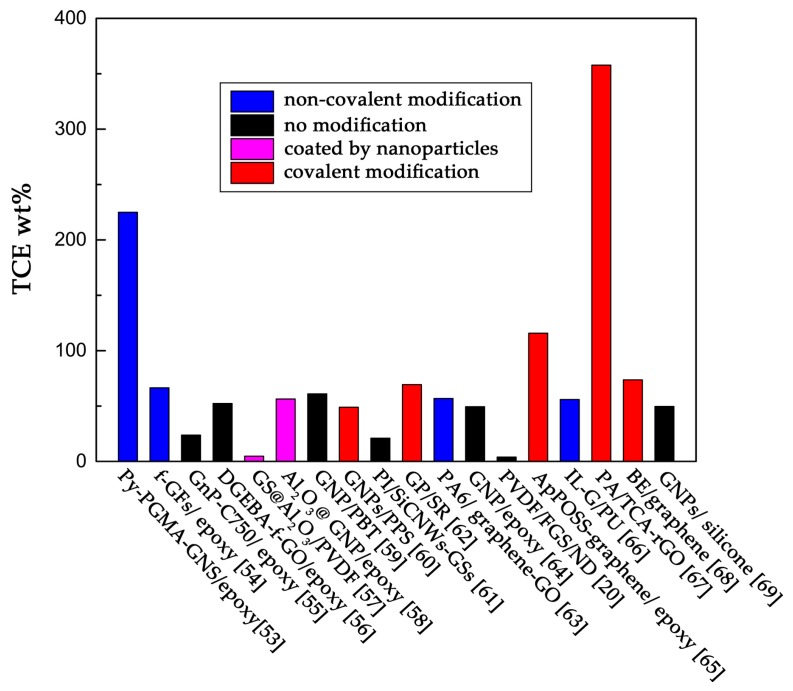
TCE wt % of composites in Table 1.

Graphene with a random orientation in the polymer matrix can be manufactured by many methods, such as solution mixing, melt mixing, and in-situ polymerization, etc. [47,48,49,50,51,52]. Table 1 lists the thermal conductivity, thermal conductivity enhancement (TCE) per wt %, preparation methods, and surface preparation methods of graphene with a random orientation. The thermal conductivity enhancement is measured by a term of TCE per wt %, which refers to the enhancement of thermal conductivity by per weight content of graphene in composites [53]. In order to find the most effective methods to enhance the thermal conductivity of composites, we compared the TCE per wt % of every composite shown in Table 1. The results are shown in Figure 4. From Figure 4, we can see that graphene is an efficient filler for enhancing the thermal conductivity of the polymer matrix. The TCE per wt % of graphene is around 50%, which means several percent of graphene can significantly increase the thermal conductivity of the composite. But the TCE per wt % of unmodified graphene is difficult to exceed 100%. However, when using graphene modified by covalent or noncovalent bonds, the TCE per wt % can be very close to 100%. When using a titanate coupling agent to modify graphene, the TCE per wt % is as high as 357.8%. The researchers believe the interfacial force between graphene and polymer has been enhanced by surface modification. This enhancement could reduce the interfacial thermal resistance and disperse graphene more uniformly [54].

### 3.2. Graphene with Specific Orientation in the Polymer Matrix

**Figure 5:**
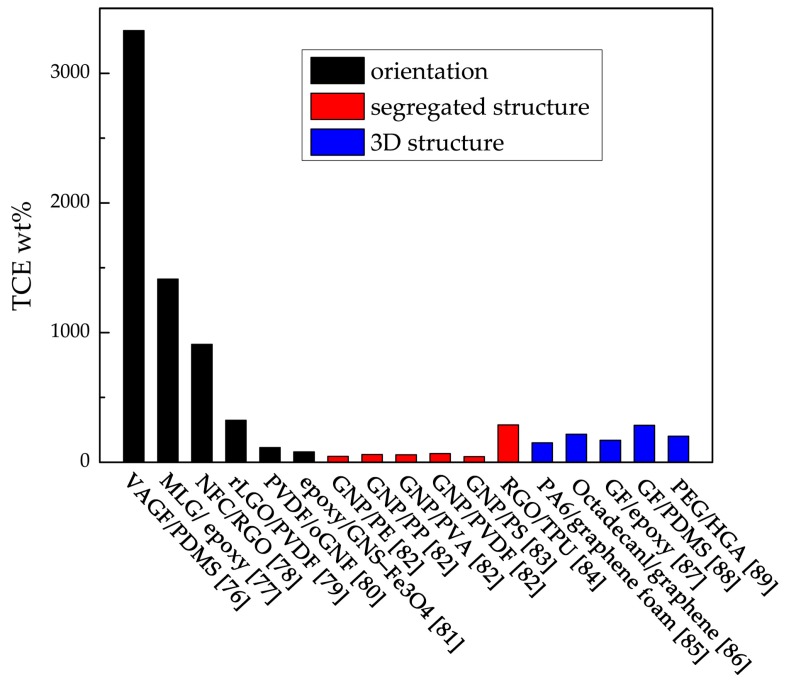
TCE wt % of composites in Table 2.

There are a variety of specific orientations of graphene in the polymer matrix, such as orientation, segregated structure, 3D structure, and so on [53,71,72,73,74,75]. Specific orientations of graphene give special properties to the composites. The thermal properties of recent studies in graphene-polymer composites are listed in Table 2, and the enhancements of each composite are compared in Figure 5. From Figure 5, it seems that the orientation and 3D structure are more efficient structures for improving the thermal conductivity of composites. By comparing Figure 4 and Figure 5, we also find that graphene with a specific orientation is more efficient than that with a random orientation. The researchers believe that this is mainly because that graphene plays the role of the thermal conduction pathway in the polymer matrix, and the heat transfers through the graphene pathway preferentially [76]. The purpose of orientation, a segregated structure, and 3D structure is establishing the thermal pathway in the polymer matrix, which can transfer heat more efficiently [77,78,79,80].

## 4. Influence Factors on Thermal Conductivity of Graphene-Polymer Composites

There are many factors affecting the thermal conductivity of graphene-polymer composites, such as the defects on graphene, the orientation of graphene in the polymer, the graphene loading, and the surface modification, etc. [3,4,5]. In this section, we mainly review the influence of the characteristics of graphene (such as the defect, morphology, number of layers, and size), the loading of graphene, the orientation of graphene in the polymer matrix, and the interface between graphene and the polymer on the thermal conductivity.

### 4.1. The Characteristics of Graphene

The characteristics of graphene have a great influence on the thermal conductivity of graphene-polymer composites [80,91,92,93]. Hoda et al. investigated the thermal conductivity of graphene as a function of the density of defects. Graphene was suspended over ~7.5 μm size square holes and the optothermal Raman technique was employed to measure the thermal conductivity of graphene in air. They found that the thermal conductivity of suspended graphene decreased from ~1.8 × 10^3^ W m^−1^ K^−1^ to ~4.0 × 10^2^ W m^−1^ K^−1^ near room temperature as the density of defects changed from 2.0 × 10^10^ cm^−2^ to 1.8 × 10^11^ cm^−2^ [94]. Xin et al. employed a high temperature to obtain defect-free graphene and investigated the thermal conductivities of polymer composites filled with graphene of different defect contents. The graphene annealing at 2200 °C had the least amount of defects, and the composite filled with it had the highest thermal conductivity, reaching 3.55 W m^−1^ K^−1^. This is because the high-temperature annealing heals defects and removes oxygen functional groups on graphene, thus reducing the phonon scattering centers [95]. The morphology of graphene also has an influence on the thermal conductivity of composites. Chu et al. pointed out that when using graphene nanoplates with more wrinkles as a filler, the composites will exhibit lower thermal conductivity. The reason for this is that the waviness of GNPs significantly affects the intrinsic characteristics of GNPs (such as thermal conductivity, aspect ratio) and the interfacial phonon coupling behavior between GNPs and polymers [96]. Kim et al. investigated the effects of the graphene layer and size on the thermal conductivity of composites and found that the thermal conductivity across the graphene/epoxy interface increases when increasing the number of graphene layers [97]. Kim et al. prepared composites filled with graphene of varied sizes and thicknesses. A similar result is found that a larger size and thickness of the graphene nanoplatelets results in an effective improvement in the thermal conductivity and heat dissipation ability of the composite [98].

### 4.2. The Loading of Graphene

The loading of graphene exerts a significant effect on the electrical and thermal conductivity of the composites [22,99,100]. It is found that there is a critical loading (percolation threshold) of graphene when the conductive composite is prepared. When the loading exceeds this value, the electrical conductivity of the composite material is improved significantly. However, it is difficult to determine whether there is a percolation threshold phenomenon in thermally conductive composites. Khan et al. researched the thermal conductivity of graphene sheets-epoxy composites. The thermal conductivity increases with increasing graphene loading and there is no percolation threshold [101]. Fazel investigated the thermal conductivity of graphene/1-octadecanol (stearyl alcohol) composites and reported a similar finding [22]. Michael et al. found strong evidence for the existence of a thermal percolation threshold in graphene nanoplatelets (GnPs)-polymer composites. Below the percolation threshold (loading < 0.17), the polymer mediates between adjacent GnPs and the GnP cannot make sufficient contact, resulting in gaps. Above the percolation threshold (loading > 0.17), there is a sharp rise in the thermal conductivity, which means that direct GnP-GnP contacts have been formed [23]. Li et al. also found a similar phenomenon in graphene-epoxy composite [102].

### 4.3. The Orientation of Graphene in the Polymer Matrix

**Figure 6:**
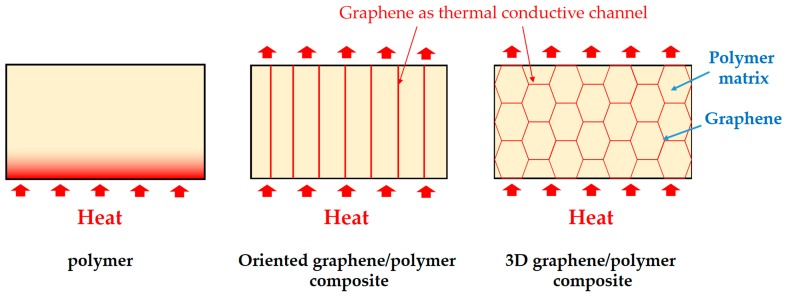
Schematic of thermal conductance in a polymer, oriented graphene/polymer composite, and 3D graphene/polymer composite.

Many researchers believe that graphene with a specific orientation, like orientation and 3D structure, is much better than graphene with a random orientation when preparing thermal conductive composites [76,77,78,79,81,82,83,84,85,86,87,88,89,90]. Zhang et al. poured polydimethylsiloxane (PDMS) into a vertically aligned graphene film (VAGF) to manufacture a high-orientation graphene-polymer composite. The thermal conductivity of this composite was as high as 614.85 Wm^−1^ K^−1^, which is higher than copper at room temperature. It is claimed that this dramatic enhancement is attributed to the rapid and effective heat-transfer path formed by orientated graphene [76]. Zhao et al. prepared a GF/PDMS composite with a thermal conductivity of 0.56 W m^−1^ K^−1^, 20% higher than that of GS/PDMS composite at the loading of 0.7 wt %. They believe that the unique interconnected structure of GF acts as an efficient thermal pathway in the polymer matrix [90]. Figure 6. is the schematic of thermal conductance in a polymer, oriented graphene/polymer composite, and 3D graphene/polymer composite.

### 4.4. The Interface between Graphene and the Polymer

It is considered that the interface between graphene and the polymer plays an important role in thermal conductive composites. Since phonons are the main form of thermal conductance in graphene-polymer composites, bad coupling in vibration modes at the graphene-polymer interface will generate huge interfacial thermal resistance. Chemical bonding between graphene and the polymer can efficiently decrease the phonon scattering at the interface and reduce interfacial thermal resistance [69]. Gao et al. investigated the influence of surface-grafted polymer chains on the thermal conductivity of a graphene-polyamide-6,6 nanocomposite. It was found that the through-plane interfacial thermal conductivity is proportional to the grafting density. Meanwhile, it first rises and then saturates as the grafting length increases. However, the in-plane thermal conductivity of graphene decreases rapidly as the grafting density increases. There is a maximum thermal conductivity of the composite because of these two competing factors [103]. Wang et al. studied the interfacial thermal resistance for polymer composites reinforced by various covalently functionalized graphene using molecular dynamics simulations. Among the various functional groups, like methyl, phenyl, butyl, formyl, carboxyl, amines, and hydroxyl, butyl is found to be the most effective one in reducing the interfacial thermal resistance [104]. Eslami et al. investigated the heat transport between graphene and polyamide-6,6 oligomers. They found that well-organized (chain stretching) polymer layers between the graphene show an interesting anisotropic heat conduction. The heat conduction in the parallel direction to the graphene surface is higher than that in the perpendicular direction [105,106].

## 5. Applications of Graphene-Polymer Composites in Thermal Engineering

Nowadays, with the improving demand in emerging industries, thermal conductive materials with novel properties are widely required [107,108,109]. Compared with other thermal conductive materials (metal, ceramics, carbon-related materials), polymer matrix composites have many outstanding properties, such as being lightweight and easy to process, and exhibiting good corrosion resistance and vibration damping, etc. In this section, some emerging applications of graphene-polymer composites are listed, such as electronic packaging, batteries, and energy storage.

### 5.1. Electronic Packaging

**Figure 7:**
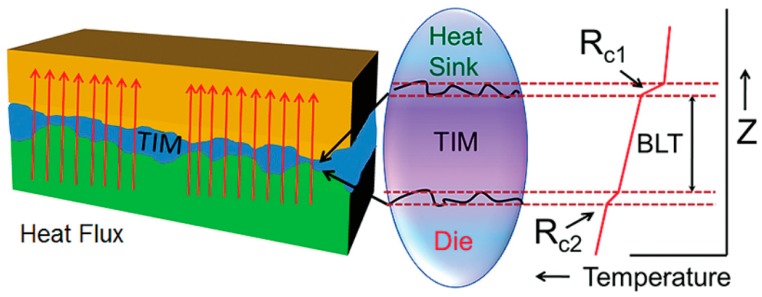
Schematic illustrating the action of thermal interface material, which fills the gaps between two contacting surfaces and conducts the heat produced by electronic drives [101]. (Copyright (2012) the American Chemical Society).

In the electronic industry, thermal management has been a serious challenge because of the miniaturization and functionalization of electronic devices. To control the temperature of all components in devices, an effective thermal conductive path must be used [47]. Thermal interface materials (TIM) are used to provide an effective heat conduction path between the two solid surfaces due to their ability to conform to rough surfaces and high thermal conductivity [110]. Figure 7 is a schematic diagram of TIM [101]. The international technology roadmap for high-performance chips at 14 nm is a power density greater than 100 W/cm^2^ and junction-to-ambient thermal resistance of less than 0.2 °C/W [2]. There is a need for TIM to dissipate heat when the chip is operating. However, the thermal conductivity of commercial TIM is relatively low as most of them are less than 5 W m^−1^ K^−1^. Employing graphene to prepare a thermal conductive material as TIM has been attracting a lot of attention.

### 5.2. Thermal Energy Storage

The effort towards thermal energy storage has been intensified over the past years. Thermal conductivity is an important parameter in thermal energy storage materials, which significantly influences the rate of heat storage and extraction [111]. Therefore, graphene and graphene derivatives are used as thermal conductive carriers to improve the thermal conductivity of thermal energy storage materials. Ji et al. embedded continuous ultrathin-graphite foams (UGFs) in phase change materials to manufacture a composite, and improved the thermal conductivity by 18 times [112]. Mehrali et al. prepared phase change materials by the vacuum impregnation of paraffin within graphene oxide, and the maximum energy storage value was 64.89 kJ/kg [113]. A phase change material consisting of graphene aerogel and octadecanoic acid was produced by Zhong et al. When the loading of GA reached 20 vol %, the thermal conductivity of this composite achieved 2.635 W/m^−1^ K^−1^, which is about 14 times that of the OA [114].

### 5.3. Batteries

As batteries have become more powerful in recent years, thermal management has turned into a special issue in the battery system. When a battery is used at a high charging/discharging rate, the rate of heat generation may exceed the rate of heat dissipation. In this situation, the battery may be inefficient or even catch fire. A thermal management system is needed to maintain the battery pack at an optimum temperature. Khan et al. incorporated 8 wt % graphene nanoflake in polyacrylonitrile (PAN) fiber separators, and the thermal conductivity increased from 3.5 to 8.5 W m^−1^ K^−1^. They think Lithium-ion batteries have become the major source of power for portable electronic devices. Separators are one of the major components of these batteries, and the improvement of thermal conductivity in separators is an option for long-lasting Li-ion battery fabrications [115]. Hallaj et al. presented a novel thermal management system and investigated it for electric vehicle applications. They think it is important to manage the heat in a battery under both cold and hot conditions [116].

## 6. Conclusions

In this paper, we have reviewed the graphene-polymer thermal conductive material in recent years. The thermal conductive mechanisms in graphene, polymers, and their composites have been discussed. The recent advances on thermal conductivity of graphene-polymer composites have also been reviewed. Furthermore, we have discussed the factors influencing the thermal conductivity of graphene-polymer composites, such as the characteristics, the loading, the orientation of graphene, and the interface. Finally, the applications of thermal conductive graphene-polymer composites have been demonstrated. This review reveals the relationship between thermal conductive mechanisms and properties and also provides guidance on the preparation of composites with high thermal conductivity.

## Figures and Tables

**Table 1:** Thermal conductivity of polymer composites filled with graphene and graphene-related materials with a random orientation.

Sample	Graphene content (wt %)	Thermal conductivity (W m^−1^ k^−1^)	Thermal conductivity enhancement (TCE) per wt %	Preparation method	Surface preparation methods
Py-PGMA-GNS/epoxy [55]	3.8	1.91	225%	In-situ polymerization	Non-covalent modification
f-GFs/epoxy [56]	10	1.53	66.5%	In-situ polymerization	Non-covalent modification
GnP-C750/epoxy [57]	5	0.45	23.8%	In-situ polymerization	no
DGEBA-f-GO/epoxy [58]	4.64 ^1^	0.72	52.3%	In-situ polymerization	no
GS@Al2O3/PVDF [59]	40	0.586	4.8%	solution mixing	Coated by alumina nanoparticals
Al2O3@ GNP/epoxy [60]	12	1.49	56.4%	solution mixing	Coated by alumina
GNP/PBT [61]	20	1.98	61%	In-situ polymerization	no
GNPs/PPS [62]	37.8 ^1^	4.414	49%	melt mixing	Covalent modification
PI/SiCNWs-GSs [63]	7	0.577	21%	solution mixing	no
GP/SR [54]	0.72	0.3	69.4%	mechanical blending	Covalent modification
PA6/graphene-GO [64]	10	2.14	56.9%	In-situ polymerization	Non-covalent modification
GNP/epoxy [65]	25	2.67	49.4%	solution mixing	no
PVDF/FGS/ND [20]	45	0.66	3.9%	solution mixing	no
ApPOSS-graphene/epoxy [66]	0.5	0.348	115.8%	solution mixing	Covalent modification
IL-G/PU [67]	0.608	0.3012	55.9%	In-situ polymerization	Non-covalent modification
PA/TCA-rGO [68]	5	5.1	357.8%	melt mixing	Covalent modification
BE/graphene [69]	2.5	0.542	73.7%	solution mixing	Covalent modification
GNPs/silicone [70]	16	~2.6	49.7%	In-situ polymerization	no

^1^ volume fraction was converted into weight fraction.

**Table 2:** Thermal conductivity of polymer composites filled with graphene and graphene-related materials with a specific orientation.

Sample	Graphene content (wt %)	Thermal conductivity (W m^−1^ k^−1^)	Thermal conductivity enhancement (TCE) per wt %	Specific orientation of graphene
VAGF/PDMS [76]	92.3	614.85	3329%	orientation
MLG/epoxy [77]	11.8	33.54	1412.7%	orientation
NFC/RGO [81]	1	12.6	910%	orientation
rLGO/PVDF [82]	27.2	19.5	~323.5%	orientation
PVDF/oGNF [83]	~36.8	~10	~113.2%	orientation
epoxy/GNS–Fe_3_O_4_ [84]	~1.74	~0.6	~79.9%	orientation
GNP/PE [85]	10	1.84	45.7%	segregated structure
GNP/PP [85]	10	1.53	59.5%	segregated structure
GNP/PVA [85]	10	1.43	58%	segregated structure
GNP/PVDF [85]	10	1.47	67.3%	segregated structure
GNP/PS [86]	~9.2	~0.9	43.3%	segregated structure
RGO/TPU [87]	1.04	0.8	288%	segregated structure
PA6/graphene foam [78]	2	0.847	150%	3D structure
Octadecanl/graphene [88]	12	5.92	216%	3D structure
GF/epoxy [89]	5	1.52	170%	3D structure
GF/PDMS [90]	0.7	0.56	285%	3D structure
PEG/HGA [79]	1.8	1.43	200.6%	3D structure

## Abbreviations

Py-PGMA-GNS/epoxy	Pyrene-end poly(glycidyl methacrylate)-graphene nanosheet/epoxy composite
f-GFs/epoxy	Non-covalently functionalized graphene flakes/epoxy composite
GnP-C750/epoxy	Graphene nanoplatelets (sizes < 1 μm)/epoxy composite
DGEBA-f-GO/epoxy	Diglycidyl ether of bisphenol-A functionalized graphene oxide/epoxy composite
GS@Al_2_O_3_/PVDF	Alumina-coated graphene sheet/poly(vinylidene fluoride) composite
Al_2_O_3_@GNP/epoxy	Alumina nanoparticles decorated graphene nanoplatelets/epoxy composite
GNP/PBT	Graphene nanoplatelet/polybutylene terephthalate composite
GNPs/PPS	Graphene nanoplatelets/polyphenylene sulfide composite
PI/SiCNWs-GSs	Polyimide/SiC nanowires grown on graphene sheets composite
GP/SR	Graphene/silicone rubber
PA_6_/graphene-GO	Polyamide-6/graphene-graphene oxide composite
GNP/epoxy	Graphene nanoplatelets/epoxy composite
PVDF/FGS/ND	Poly(vinylidene fluoride)/functionalized graphene sheets/nanodiamonds composite
ApPOSS-graphene/epoxy	Aminopropylisobutyl polyhedral oligomeric silsesquioxane grafted graphene/epoxy composite
IL-G/PU	1-allyl-methylimidazolium chloride ionic liquid modified graphene/polyurethane composite
PA/TCA-rGO	Titanate coupling agent modified reduced graphene/polyamide composite
BE/graphene	Bio-based polyester/graphene composite
GNPs/silicone	Graphene nanoplatelets/silicone composite
VAGF/PDMS	Vertically aligned graphene film/polydimethylsiloxane
MLG/epoxy	Multilayer graphene/epoxy composite
NFC/RGO	Nanofibrillated cellulose/epoxy composite
rLGO/PVDF	Highly self-aligned large-area reduced graphene oxide/poly (vinylidene fluoride-co-hexafluoropropylene) composite
PVDF/oGNF	Oriented graphene nanoflake/poly(vinylidene fluoride) (PVDF) composite
epoxy/GNS–Fe_3_O_4_	Epoxy/graphene nanosheets-Fe_3_O_4_
GNP/PE	Graphene nanoplatelets/polyethylene
GNP/PP	Graphene nanoplatelets/polypropylene
GNP/PVA	Graphene nanoplatelets/poly(vinyl alcohol)
GNP/PVDF	Graphene nanoplatelets/poly(vinylideneuoride)
GNP/PS	Graphene nanoplates/polystyrene; RGO/TPU
RGO/TPU	Reduced graphene oxide/thermoplastic polyurethane
PA_6_/graphene foam	Polyamide-6/graphene foam
GF/epoxy	Graphene foams/epoxy
GF/PDMS	Graphene foam/polydimethylsiloxane
PEG/HGA	Polyethylene glycol/hybrid graphene aerogels
